# Recurrence of Prostate Cancer with Cutaneous Metastasis after Radical Prostatectomy

**DOI:** 10.1155/2015/825175

**Published:** 2015-03-12

**Authors:** Parth Patel, Jay Patel, Sameer Siddiqui

**Affiliations:** ^1^St. Louis University School of Medicine, St. Louis, MO 63103, USA; ^2^Metropolitan Urological Specialists, St. Louis, MO 63122, USA; ^3^Division of Urology, St. Louis University Hospital, St. Louis, MO 63103, USA

## Abstract

While cutaneous metastases are already extremely rare in primary metastatic prostatic adenocarcinoma, cutaneous manifestations in recurrent prostate cancer have rarely been described prior to this report. Here we present the case report of a 93-year-old male who underwent radical prostatectomy but eventually suffered from a previously undescribed recurrence of prostatic adenocarcinoma with distant cutaneous metastases to proximal right lower leg.

## 1. Introduction

Adenocarcinoma of the prostate is the most commonly diagnosed nondermatological malignancy among men and a leading cause of cancer-related mortality, second only to lung cancer. Despite the high incidence and prevalence of prostate cancer in society, distant cutaneous metastases tend to occur exceptionally infrequently—reported at <0.1% in primary disease [1]. While cutaneous metastases are already extremely rare in primary metastatic prostatic adenocarcinoma, cutaneous manifestations in recurrent prostate cancer have rarely been described prior to this report [2–4]. Of note, all of those patients had cutaneous metastases to trocar sites within one year after laparoscopic resection, suggesting surgical seeding as the primary etiology. Here we present the case report of a patient who underwent radical prostatectomy but eventually suffered from a previously undescribed recurrence of prostatic adenocarcinoma with distant cutaneous metastases.

## 2. Case Report

**Figure 1:**
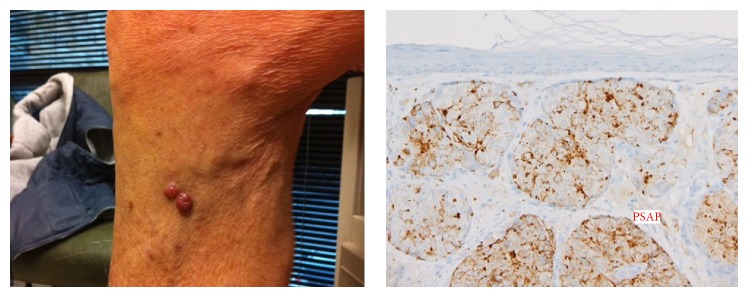
Gross image of cutaneous metastasis and histology of shave biopsy, strongly positive throughout the cytoplasm for PSAP.

**Figure 2:**
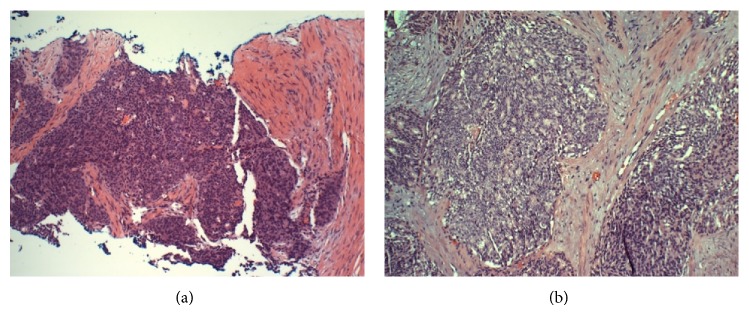
Biopsies of corpus cavernosum (a) and bladder neck mass (b), both demonstrating poorly differentiated prostatic adenocarcinoma.

A 93-year-old man presented with gross hematuria and a red, translucent papule on his proximal right lower leg (Figure 1). Twenty years ago he had ultrasound-guided needle biopsies after a suspicious digital rectal exam, which yielded a Gleason score of 6 (3+3), and he underwent a radical prostatectomy with negative regional lymph nodes. Perioperative laboratory records from the operation were not available. The patient remained under the supervision of his primary care physician, but his PSA levels had not been followed up for several years. At the time of this presentation, his PSA level was measured at 415 ng/mL, the papule was biopsied, and an abdominopelvic CT scan was ordered. Tissue obtained via shave biopsy was weakly positive at the luminal zones for PSA and strongly positive throughout the cytoplasm for PSAP, which proved the lesions to be metastatic prostate adenocarcinoma (Figure 1). CT scan revealed multiple hepatic lesions, which were presumed to be metastatic, without any evidence of retroperitoneal nodal metastases. During cystoscopic evaluation of the patient's gross hematuria, a friable bladder neck mass was resected with loop electrocautery, and penile corporal tissue was also biopsied—both of which showed poorly differentiated prostatic adenocarcinoma (Figure 2). The patient's gross hematuria resolved after the bladder neck mass was resected, but he did suffer from priapism secondary to corporal invasion. Given his age and extent of disease, the patient was managed for palliation with antiandrogen therapy—leuprolide and bicalutamide—for systemic control and focused radiation for local control. Three months after the initiation of therapy, his PSA was measured at 14 ng/mL, and no further disease progression was noted.

## 3. Discussion and Analysis

Next to early-stage primary dermatological neoplasms, prostate cancer remains one of the most curable malignancies, with encouraging recurrence-free five-year survival rates greater than 80% [5]. Most patients respond well to local curative therapy, but the rate of recurrence is fairly significant: 23% for patients who have undergone radical prostatectomy [6]. While metastases to bone are not uncommon in advanced primary or recurrent disease, cutaneous metastases are extremely rare. These types of cutaneous manifestations have been hypothesized to result from direct extension of the malignancy, seeding during surgical resection, and hematogenous or lymphatic spread. Based on a review of the literature in the English language, there are reports of 37 patients with cutaneous metastases. In these patients metastatic lesions from primary prostate cancer have been found in the inguinal region, umbilical region, chest, back, head, and face, and lesions that can be associated with recurrent disease have only been found at trocar sites after surgical intervention [7]. In contrast, our patient presented nearly two decades after having radical prostatectomy with significant systemic involvement and distant cutaneous lesions to the right lower leg—a truly unique presentation.

Cutaneous manifestations are associated with a poor prognosis because of the presumed systemic involvement required to develop such overt metastatic disease. The mean survival time after diagnosis of cutaneous metastasis has been calculated at 7 months [8]. Because most of these patients already have advanced disease, no trials have been conducted to evaluate or compare the various treatment methodologies. Providers have historically taken a conservative approach in the management of these patients, with most patients receiving palliative care and local radiation therapy, in attempts to treat symptoms and improve the patients' quality of life. Of patients receiving more aggressive treatment for cutaneous metastases, chemotherapeutic agents such as capecitabine, cyclophosphamide, cisplatin, and etoposide have been utilized without much improvement [9, 10]. More encouraging results have been achieved—with almost complete resolution of cutaneous lesions—in patients, including ours, who have received androgen deprivation therapy with leuprolide and bicalutamide [7, 9]. Given the aggressive nature of disseminated prostate cancer and the dismal prognosis of patients with cutaneous metastases, the management of these patients remains at the discretion of their providers.
